# Wikipedia: A Key Tool for Global Public Health Promotion

**DOI:** 10.2196/jmir.1589

**Published:** 2011-01-31

**Authors:** James M Heilman, Eckhard Kemmann, Michael Bonert, Anwesh Chatterjee, Brent Ragar, Graham M Beards, David J Iberri, Matthew Harvey, Brendan Thomas, Wouter Stomp, Michael F Martone, Daniel J Lodge, Andrea Vondracek, Jacob F de Wolff, Casimir Liber, Samir C Grover, Tim J Vickers, Bertalan Meskó, Michaël R Laurent

**Affiliations:** ^22^Department of Internal MedicineUniversity Hospitals LeuvenLeuvenBelgium; ^21^Medical School and Health Science CenterUniversity of DebrecenDebrecenHungary; ^20^Department of Molecular MicrobiologySchool of MedicineWashington UniversitySt. Louis, MOUnited States; ^19^Division of GastroenterologyUniversity of TorontoToronto, ONCanada; ^18^School of PsychiatryCollege of MedicineUniversity of New South WalesSydney, NSWAustralia; ^17^Department of PsychiatryBankstown Health ServiceSydney, NSWAustralia; ^16^Department of Acute MedicineUniversity College HospitalLondonUnited Kingdom; ^15^Department of ImmunologySchool of Medicine and National Jewish HealthUniversity of ColoradoDenver, COUnited States; ^14^Department of Cardiac SurgeryUniversity of TorontoToronto, ONCanada; ^13^Medical CollegeRush UniversityChicago, ILUnited States; ^12^Department of RadiologyLeiden University Medical CenterLeidenNetherlands; ^11^Department of DermatologyUniversity of IllinoisChicago, ILUnited States; ^10^Division of Cellular and Molecular PathologySchool of MedicineUniversity of QueenslandBrisbane, QldAustralia; ^9^Anatomical Pathology DepartmentPathology QueenslandRoyal Brisbane and Women’s HospitalBrisbane, QldAustralia; ^8^College of MedicineUniversity of VermontBurlington, VTUnited States; ^7^MicrobiologyWalsall Manor HospitalWalsallUnited Kingdom; ^6^Departments of Internal Medicine and PediatricsMassachusetts General HospitalHarvard Medical SchoolBoston, MAUnited States; ^5^Department of Respiratory MedicinePoole General HospitalPooleUnited Kingdom; ^4^Department of Laboratory Medicine and PathobiologyUniversity of TorontoToronto, ONCanada; ^3^Department of Obstetrics, Gynecology and Reproductive SciencesRobert Wood Johnson Medical SchoolUniversity of Medicine and Dentistry of New JerseyNew Brunswick, NJUnited States; ^2^Department of Emergency MedicineMoose Jaw Union HospitalMoose Jaw, SKCanada; ^1^College of MedicineUniversity of SaskatchewanSaskatoon, SKCanada

**Keywords:** Internet, Wikipedia, public health, health information, knowledge dissemination, patient education, medical education

## Abstract

The Internet has become an important health information resource for patients and the general public. Wikipedia, a collaboratively written Web-based encyclopedia, has become the dominant online reference work. It is usually among the top results of search engine queries, including when medical information is sought. Since April 2004, editors have formed a group called WikiProject Medicine to coordinate and discuss the English-language Wikipedia’s medical content. This paper, written by members of the WikiProject Medicine, discusses the intricacies, strengths, and weaknesses of Wikipedia as a source of health information and compares it with other medical wikis. Medical professionals, their societies, patient groups, and institutions can help improve Wikipedia’s health-related entries. Several examples of partnerships already show that there is enthusiasm to strengthen Wikipedia’s biomedical content. Given its unique global reach, we believe its possibilities for use as a tool for worldwide health promotion are underestimated. We invite the medical community to join in editing Wikipedia, with the goal of providing people with free access to reliable, understandable, and up-to-date health information.

## Introduction

The Internet allows unprecedented opportunities for patients and the general public to retrieve health information from across the globe. Surveys have shown that online health information retrieval is both common and increasing [1-4]. Population-based studies have shown that 61% of American and 52% of European citizens have consulted the Internet for health-related information on at least one occasion [1,4]. Similarly, numerous cross-sectional surveys in patient populations have shown variable but considerable rates of eHealth activities [5-10]. Physicians frequently report that patients have searched the Internet regarding health issues [11,12], although patients do not always discuss these online activities with their doctors [13,14]. Among American e-patients, 44% said this information had a minor impact and 13% said it had a major impact on their decisions about health care [4].

Websites offering medical information differ widely in their quality [15]. While physicians should reasonably view trustworthy information as useful, some have voiced concerns that Internet information may undermine their authority and lead to self-treatment [13]. Furthermore, incorrect medical information could result in patient harm. Indeed, about 3% of users of health care information feel that they or someone they know has been seriously harmed by Web-based information [4]. A potential solution for these drawbacks is that physicians direct online health information seekers to quality resources. This so-called Internet prescription has been evaluated in a few randomized trials, which showed that it increases use of the recommended websites [16-18]. Despite concerns over the quality of health websites, the 2005 Health On the Net survey found that medical Internet users value information availability and ease-of-finding more than accuracy and trustworthiness [13].

General search engines, of which Google is the market leader in Western countries, appear to be the most common starting point for laypeople seeking health information, despite the existence of eHealth quality labels and special search engines to explore health information [4,10,13,19,20]. Search engines commonly lead seekers to Wikipedia [21]. In the 2009 Pew Internet survey on health information, 53% of e-patients had consulted Wikipedia (not necessarily related to health information) [4]. This paper examines the role of Wikipedia as a provider of online health information.

## Wikipedia: An Internet Heavyweight

### Core Features of Wikipedia

Wikipedia is a freely accessible, multilingual, Web-based, free-content encyclopedia that is written collaboratively by volunteers from countries around the world. It is the largest reference website and the most prominent example of a wiki, with over 3.3 million articles in English alone accrued between its inception in January 2001 and May 2010. Wikis allow anyone reading a particular page to also alter it using relatively simple editing commands. Wikipedia maintains a public record of all previous changes to improve collaboration between multiple editors. Everyone is invited to edit, with most changes appearing immediately after submission. Wikipedia is supported by a nonprofit organization, the Wikimedia Foundation, and is free of commercial interests and advertisements. It is one of the most commonly used websites on the Internet, attracting around 362 million visitors monthly as of January 2010 or 29% of global Internet users, making it the sixth most popular website on the Internet [22,23]. The multimedia content used across all Wikimedia projects is stored in a central repository (Wikimedia Commons), which hosts more than seven million freely licensed media files.

### Content Creation and Maintenance

Wikipedia’s open editorial policy is a departure from the traditional encyclopedias written exclusively by experts. Its editors often write using a pseudonym with no easy way to verify their credentials or expertise. The lack of vetting by identifiable experts has led to the critique that the editorial process favors consensus over credentials [24]. Additions to articles are judged based upon their verifiability, and information added without references may be challenged or removed. The development of Wikipedia’s articles has been described in evolutionary terms; that is, each phrase and sentence is subject to scrutiny and review over and over again, so that eventually “only the fittest” of these will survive, while unsustainable sections will be eliminated [25]. Fitness is determined by verifiability, ease of understanding, and completeness. The goal is an easy-to-read, thoroughly referenced article that is broad in scope. Such an article is less subject to major edits unless there are changes in the subject matter itself. As articles are improved, editors can nominate them for quality labels. Promotion to Good Article status requires independent review by one editor. A common next step would be Wikipedia’s peer review process, whereby an article is subjected to closer scrutiny from a broader group of editors. The highest-quality articles are Featured Articles, a label that is applied only when there is consensus that the article exemplifies Wikipedia’s best work.

Articles can be damaged in a number of ways, including deletion of information, insertion of misinformation or nonsense, use of offensive language, and addition of spam defined as advertisements or nonuseful links [26]. People who are unaware of Wikipedia’s quality control measures may find it surprising that Wikipedia’s content is not compromised more frequently. However, multiple layers of quality control are in place to prevent or revert spurious additions or removals. These include the following:

- Watchlist: People with an interest in a particular subject can be notified when edits are made to articles they are following.
- Recent changes: Volunteers judge the merits of each change throughout Wikipedia through a list of recent changes (with or without the help of vandalism-fighting software).
- Bots: A system of automated computer scripts, developed by volunteers, fixes a range of problems such as common grammatical and spelling errors, simple vandalism, and copyright violations.
- Page protection: Pages that are highly likely to attract vandalism or controversy can be partly or fully protected from editing by less-established editors.
- Edit filter: Certain edits can be prevented by built-in filters, such as removing references or large sections by new editors. This can also be applied to sensitive medical information: for example, when a filter was established to prevent removal of the Rorschach ink blots [27].
- Blocking and banning: Both anonymous and logged-in editors who demonstrate noncontributory or disruptive editing (eg, page blanking, spamming) can temporarily or permanently have their editing privileges removed.

Some of these maintenance tools (eg, page protection and blocking) are operated by trusted, established editors called administrators. Although it is impossible to guarantee the validity of every Wikipedia article, as no one person is ultimately responsible for the content, the development of an elaborate antivandalism system explains the paradox of how quality can be sustained in a radically open editing system. In one study, 42% of damaged articles were repaired within one viewing and thus had no impact, while 11% were still present after 100 viewings [26]. This shows that, while the system is surprisingly effective, there remains room for improvement.

As of June 2010, Wikipedia is experimenting with a system of Flagged Revisions or Pending Changes, whereby the edits of anonymous and new users (those with fewer than 200 edits) require a sign-off by an established editor before they are made visible. This system has been in use on the German-language Wikipedia since May 2008, and other-language Wikipedias (eg, Russian and Polish) have followed since. Another system under investigation is WikiTrust, which color codes article content that is unstable and possibly unreliable based on the credibility of content and reputation of the author [28]. Registered users can already modify their settings so that article quality information from assessments is displayed in color at the top of the article. Another proposal includes specifically protecting critical health-related information. We believe that these are examples of a trend toward more control over the editing process.

### Who Writes Wikipedia?

Wikipedia has attracted a few thousand prolific and dedicated editors plus a large number of both registered editors (>12 million) and anonymous visitors who make edits less frequently (the so-called long tail) [29,30]. About 0.1 % of editors contribute nearly half of Wikipedia’s value as measured by words read [26]. However, all contributors are needed to improve article content and quality.

### WikiProject Medicine

**Figure 1:**
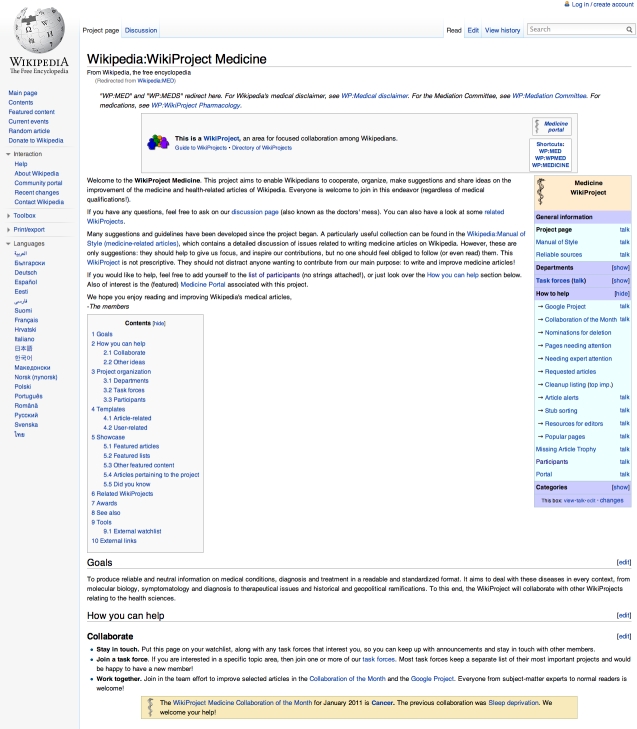
WikiProject Medicine. URL: http://en.wikipedia.org/wiki/WP:MED

**Figure 2:**
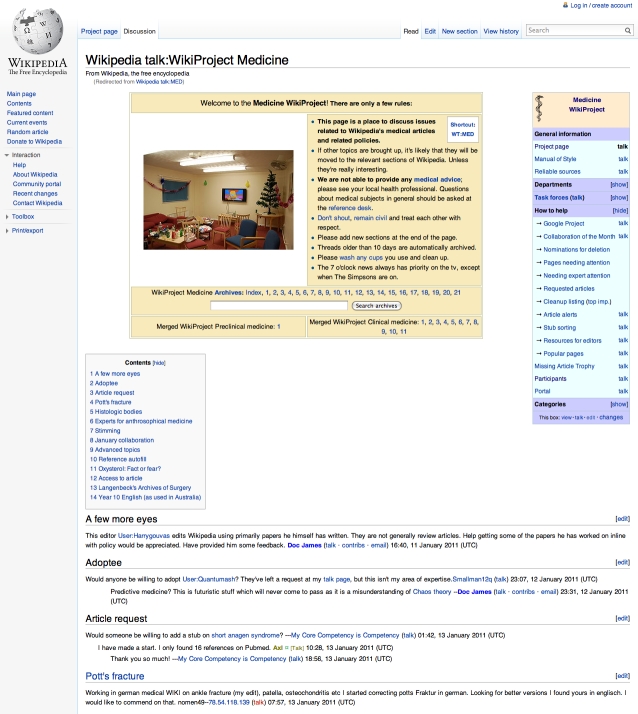
The doctor’s mess at the WikiProject Medicine. URL: http://en.wikipedia.org/wiki/WT:MED. Questions about editing medicine-related Wikipedia articles or joining WikiProject Medicine may be posted here.

Groups of editors interested in a certain field of knowledge can collaborate through so-called WikiProjects. WikiProject Medicine (Figure 1) was founded in April 2004. It has more than 200 listed participants as of 2010, many of whom discuss Wikipedia’s biomedical content at the virtual “doctor’s mess” [31] (Figure 2) (the authors of this paper are all members of the group). Membership does not require any credentials, but most members are doctors, medical students, nurses, scientists, patients, or laypeople with an interest in specific medical topics. Project members have been responsible for creating a style manual that provides specific guidance on writing health-related articles, including the naming of articles, avoidance of jargon and eponyms, and a standard outline for articles on diseases and medications (in collaboration with WikiProject Pharmacology). Another guideline drafted by WikiProject Medicine participants deals with finding and selecting high-quality references. In accordance with its guideline on verifiability, Wikipedia lends itself very well to evidence-based medicine. Notably, it automatically recognizes PubMed Identifier (PMID) codes (for example, the text “PMID 11720967” would automatically be converted into an external link to the corresponding article’s abstract in Medline).

Wikipedia articles are graded by WikiProjects according to defined quality measures, similar to peer review. Wikipedia contains more than 20,000 health-related articles and more than 6200 articles related to drugs and pharmacology (with an overlap of roughly 700 articles), based on article assessment data from WikiProject Medicine and WikiProject Pharmacology [32,33]. Other activities of WikiProject Medicine include a periodic collaboration on a specific article (the Collaboration of the Month) and Task Forces focusing on different specialty topics (eg, cardiology, dermatology).

## Wikipedia as a Source of Health Information

### A Prominent Resource

Wikipedia contains a large amount of health information, which is accessed extensively by both the lay public and health care providers. Studies have found that 70% of junior physicians use Wikipedia in a given week, while nearly 50% to 70% of practicing physicians use it as an information source in providing medical care [34-36]. The junior physicians used Wikipedia more frequently than all other websites excluding Google [34]. Of pharmacists who responded to a questionnaire, 35% admitted using it [37]. The medical articles on Wikipedia receive about 150 million page views per month, with the top 200 most-visited medical articles each receiving more than 100,000 views per month and the top 500 each receiving greater than 60,000 views per month [38]. While some of the most popular articles are of featured or good quality (eg, Asperger syndrome, schizophrenia, and tuberculosis), many other popular articles require improvement. In 2008 the English Wikipedia had the highest average search engine ranking for health terms in comparison with other health resources such as MedlinePlus, WebMD, and NHS Direct. It was ranked among the first 10 Google search hits for medical keywords obtained from various indexes in greater than 70% of cases, being first place in 25% to 33% of cases [21]. The higher a website is ranked among search engine results, the more likely it is that (inexperienced) searchers will view it, with an exponential decay after the first page of results [19,20]. With the importance of search engines such as Google for people who seek health information, we believe that Wikipedia’s global reach gives it a vast and underestimated potential as a tool for medical knowledge translation.

### Wikipedia’s Strengths and Weaknesses

Wikipedia’s approach has proven to be remarkably successful as evidenced by its scope and popularity. The main criticism focuses on the open nature of the editing process, which inherently poses risks of inaccuracies. One commentator summarized the situation as follows: “Wikipedia is both phenomenally successful and, in the eyes of some critics, fundamentally flawed” [39]. A reader can never be absolutely certain that information is not corrupted but, as we have discussed earlier, elaborate quality control mechanisms are in place, and are likely to expand in the future. Another drawback of Wikipedia is that in the intermediate-quality articles, the writing by many different editors may give articles an uneven, choppy quality [40].

Some people use Wikipedia’s articles to advance their personal beliefs, and so the encyclopedia has been criticized for hosting fringe theories, quackery, and unbalanced views [41]. When editors hold conflicting views regarding the content of an article, an elaborate process exists for dispute resolution, guided by Wikipedia’s core policies of verifiability and neutral point of view. Each article has an associated discussion page where multiple editors can coordinate their efforts and resolve any editing controversies. If this route fails, editors can request assistance from experienced editors, solicit comments from a wider part of the community, and request informal and formal mediation and, ultimately, arbitration. As Wikipedia has grown, the rate of creation of new articles and content has decreased, while levels of maintenance and indirect work (including coordination and conflict resolution) are increasing [42]. Some editors avoid editing in controversial areas, which is perfectly acceptable since plenty of noncontroversial areas need substantial improvements. Wikipedia has a strict policy against personal threats in discussion, although in extremely rare instances online editing controversies can have consequences in real life (for example, the first author of this article was investigated based on his Wikipedia editing [43]). As long as editors keep in mind their professional obligations while contributing, we believe that editing Wikipedia poses fewer dangers than social media websites, for example [44,45].

A strength of Wikipedia is its ability to be updated swiftly, whereas traditional peer-reviewed articles in rapidly evolving fields can be outdated even before they are published [46]. Prominent examples of Wikipedia’s capability to update almost instantaneously are articles on disease outbreaks, such as the 2009 influenza pandemic.

### Empirical Studies on Wikipedia’s Medical Content

Wikipedia articles have occasionally been cited in scientific articles, although this remains controversial [47]. Between 2004 and 2009, it was among the referenced works in the ISI Web of Science 263 times, while the Encyclopædia Britannica was only cited 10 times [48]. Wikipedia’s reliability has been tested in a number of studies, notably in a favorable comparison with Britannica [49]. Wikipedia articles increasingly contain references, with high impact factor medical journals such as the *New England Journal of Medicine*, *The Lancet*, the *Journal of the American Medical Association*, and the *British Medical Journal* among the 10 most frequently cited science journals in Wikipedia in 2007 [50].

Empirical studies evaluating Wikipedia’s medical content have recently started to emerge. In a study examining drug information, Medscape Drug Reference provided answers to 82.5 % of predetermined questions, while Wikipedia could answer only 40% [51]. While there were few factual errors, Wikipedia articles were often missing important information, like drug dosages, interactions, and contraindications. However, the authors failed to acknowledge that the Wikipedia style manual for drug articles specifically discourages mentioning dosages, as such information is rarely within the scope of a general encyclopedia and corruption of this information could result in serious harm. The authors did point out that drug company representatives have been caught deleting information from Wikipedia entries that make their drugs look unsafe [51]. A study that looked at Wikipedia articles pertaining to the most commonly performed inpatient surgical procedures found that, while these pages were accurate, they still had critical content omissions [52]. Another paper comparing the appropriateness of articles in Wikipedia with those in UpToDate, eMedicine, and AccessMedicine for medical student use found that Wikipedia was the easiest to use and access; however, it lacked the depth and accuracy of the other three traditional online medical resources [53]. An analysis of the suitability of Wikipedia for nursing students found that the average medical article contained 29 reputable sources [54].

A recent evaluation found Wikipedia accurate enough to include parts of it in a laboratory observations database [55]. Another Web-based study found that Wikipedia had entries on 82.8% of gastroenterological conditions selected from the *International Classification of Diseases*, 10th revision [56]. Of these articles, 65% were substantiated with at least one peer-reviewed reference, and the average number of references per article was 6.8. The median Flesch-Kincaid reading level was above high school grade (13.7 years). Another analysis presented at the 2010 Annual Meeting of the American Society of Clinical Oncology, based on 10 articles dealing with cancer, found that errors “were extremely rare on Wikipedia” (<2%) but information was less easy to understand than that in the US National Cancer Institute’s PDQ (Physician Data Query), a peer-reviewed cancer database [57]. An assessment of the scope of Wikipedia’s coverage of pathology informatics in 2010 found that 90% of terms in the Association for Pathology Informatics curriculum had a corresponding Wikipedia page. The contents of the pages were deemed comprehensive, of high quality, current, and useful for both the beginner and advanced learners [58].

The main conclusions that can be drawn from these studies are that the medical information on Wikipedia is found in articles on many topics that contain few factual errors, although the depth of individual articles and the ease of understanding need to be improved substantially. Nevertheless, Wikipedia’s medical disclaimer warns that articles may contain inaccuracies, and Wikipedia’s article on its own reliability states that it can be a valuable starting point when researching a topic, but that users should take care – as with all general reference works – to check facts and be aware that mistakes and omissions do occur.

### Comparison With Other Medical Wikis

Wikipedia is but one of many free online encyclopedias with medical content that allow user contributions. At least 70 medical wikis have been cataloged [59]. Some of them are devoted to medical specialties (such as Radiopaedia.org and WikiSurgery.com), while others deal with medicine in general (such as Ganfyd.org and Wikidoc.org). Health topics are also part of Web-based encyclopedias attempting to cover all human knowledge (such as Wikipedia and Citizendium.org). Several specialized medical wikis offer the benefit of verification of the editors’ credentials, and specific topics can be dealt with more elaborately than in a general wiki (even Wikipedia encourages moving overly specific content to dedicated wikis if it falls outside the scope of a general encyclopedia). On the other hand, being a general encyclopedia, Wikipedia has the advantage that topics indirectly related to medicine (eg, concepts of physics or chemistry underlying medicine) are presented in detail in the same encyclopedia.

To achieve sustainability and to guarantee a minimal editing rate, wikis need to establish a critical mass of contributors. A selection of wikis and competing websites is shown in Table 1, which demonstrates the unique and dominant position of Wikipedia in terms of access, breadth, and reach (note that although Google Knol is compared with other websites in this table, it is not a wiki). Nevertheless, depth and quality need improvement, as more than 80% of the 20,000 medical articles are still in the earliest developmental stage (Stub- or Start-class articles on the Project Assessment scale), while only 90 articles are Good Articles and 70 are Featured Articles or Lists, approximately.

**Table 1:** Comparison of selected wikis containing medical information

Encyclopedia	Year	Content license^a^	Scope	Number of English articles	Ranking (percentage) of global Internet traffic^b^	Contributors	Number of editors	Languages
Wikipedia.org	2001	cc-by-sa	General	>3.3 million; >20,000 medical, >6000 drug related	6th (13.0%)	Anyone	>12 million registered	271
Radiopaedia.org	2005	cc-by-nc-sa	Radiology	~4000	642,225 (0.00022%)	Registered users	3800	1
Wikidoc.org	2005	cc-by-sa	Medicine	~71,500^c^	191,463 (0.00105%)	Registered users	>2000	8
Ganfyd.org	2005	medical-by-nc-sa^d^	Medicine	>8000	665,248 (0.00027%)	Medical	450	1
Askdrwiki.com	2006	cc-by-nc-sa	Medicine	>2000	1,199,394 (0.00014%)	Medical	1100	1
Citizendium.org	2006	cc-by-sa	General	~13,900	52,188 (0.00209%)	Registered users	>9000	1
Knol.google.com	2008	As per contributor	General	>100,000; >5900 medical	Unknown	Registered users	Unknown	12
Medpedia.com	2009	cc-by-sa	Medicine	>10,000	43,869 (0.00233%)	Medical	~2600	1

^a^ Abbreviations used: cc = Creative Commons license, by = attribution required, nc = non commercial use, sa = share-alike, reproduction under the same license.

^b^ Visitors between March and June 2010, according to Alexa, Inc.

^c^ Many of Wikidoc’s articles are derived from Wikipedia.

^d^ Ganfyd has its own specific license, which does not allow altering, transforming, or building upon the content unless the editor is a registered medical practitioner within the United Kingdom, Australia, New Zealand, Canada, Switzerland, or the United States.

### A Unified Platform for Disseminating Medical Knowledge

Traditionally the medical community has relied on an authoritarian “push” model to disseminate information. Yet with the rapid growth of the Internet as a source of health information, the question is not how we can encourage people to use a particular set of reliable health resources (as with an Internet prescription), but how we can best provide the global community with accessible, free, up-to-date, easy-to-understand, and comprehensive information. Wikipedia already has a worldwide audience for disseminating health information and its format has proven to foster mass collaboration. Why not adopt Wikipedia as the platform for the global medical knowledge database proposed at the dawn of the Medicine 2.0 age [60]? Instead of each creating their own health information website, patient groups, foundations, charities, professional societies, hospitals, and medical journals could all participate in and contribute to a reference work where most are likely to look first. To quote Peter Frishauf, the founder of Medscape [46]:

In Wikipedia you read one living article written by many, continually updated by many. Who needs 50 articles on avian flu when one will do?

Increased participation of the medical community is important to improve article quality and will benefit the larger audience of e-patients and health care providers. Physicians will benefit as they can use the free-content articles for patient education. Non-English-speaking patients can be given information in their native languages if these pages are available and satisfactory, or the English article could be translated into one of the more than 250 languages in which Wikipedia exists.

## A Call to Action

### Why Contribute?

Of American physicians who use Wikipedia about 10% edited one or more articles [35]. A study in Germany looked at motives for editing Wikipedia and determined that participants had a high degree of intrinsic motivation, enjoyed their autonomy when contributing, found their work to be of significance, and accepted the time and effort needed to invest in this activity to derive these benefits in return [61]. Studies have not examined why health professionals would participate in editing and organizing medical articles on Wikipedia. This requires much time and effort and, contrary to scientific publications, Wikipedia articles have no direct authorship, thus the prestige of authorship so typical for scientific articles is not attained. An attempt at recognition of authorship can be found more explicitly in competing websites such as Google Knol or Medpedia. However, the high search engine ranking of Wikipedia led Peter Frishauf to conclude [46]:

For writers, Wikipedia offers neither authorship, recognition, reward, nor punishment. Articles aren’t indexed, but with Google and Yahoo!, who needs it? The motivation for writing is love of information and a desire to share it.

We propose that physicians may contribute to Wikipedia for several reasons:

- It may be personally satisfying to provide an important educational service for individuals looking for health information, and to see articles grow that one created or improved.
- While not having a high scientific impact, Wikipedia’s articles have a high social impact due to its broad readership. In the experience of the authors, a newly created article can often be found among the top Google results within a day, often outperforming review articles in highly regarded medical journals.
- Editing or adding information helps contributing students or professionals master the subject matter and learn more about the evidence underpinning it.
- Translating complex ideas into accessible concepts and language is an interesting intellectual challenge, which can help in everyday nontechnical communication with patients.
- Writing for Wikipedia teaches modern online communication.
- WikiProject Medicine offers participation and recognition in a Web-based international community.

Wikipedia can be used as an education opportunity for both students and physicians. Medical schools should challenge their students not only to read Wikipedia’s articles critically, but also to rewrite, discuss, critique, and improve them. The experiences of a group of graduate students editing Wikipedia was described in a 2009 publication as “extremely valuable as an exercise in critical thinking and communication skills” [62].

Several options exist to create direct incentives for health professionals and biomedical scientists to contribute to Wikipedia. WikiProject Medicine members are applying to get recognition as a continuing medical education (CME) opportunity, so that professionals could get credits for editing medical content. Authorship of Wikipedia could also be counted similarly to a scientific publication for people requesting grants or funding. Scientific journals could couple traditional publishing with contributions to Wikipedia. An example of this is the scientific journal *RNA Biology*, which requires authors on a series of review articles on RNA families to also update or create the relevant Wikipedia entry [63]. Similarly, medical journals could enhance their “social impact factor” [64] by requiring submitting authors to review a related Wikipedia entry, or by releasing a key figure or clinical image under a free-content license so that it can be incorporated into Wikipedia.

### Examples of Collaborations

Recently the US National Institutes of Health have started an initiative to encourage its scientists to contribute to Wikipedia. This is a recognition of Wikipedia’s global reach and an effort to strengthen Wikipedia’s scientific underpinnings [65]. A collaboration of the RNA WikiProject with the Rfam database, a collection of RNA families, has allowed mutual data exchange and community annotation of the Rfam database [66]. Google.org, the philanthropic arm of Google that uses information and technology to address global challenges in areas such as health, poverty, and the environment, is reviewing and translating medical articles [67]. Wikipedia’s open access model makes it ideally placed for health education in developing and developed countries alike. For example, Wikipedia articles are used for humanitarian purposes in the One Laptop per Child Project and the CD selection for SOS Children UK, and so its medical articles could assist in providing health care information for all [68-70].

## Conclusion

Wikipedia’s goal is to give the world free access to the sum of all human knowledge. Pursuing this, Wikipedia has evolved into an important medical resource for the general public, students, and health care professionals. While it has attracted a sizable number of experts that are enlarging its medical content, its potential to improve health may not yet be fully appreciated. While some authors have called for a variant of Wikipedia for medicine [46,71], many wikis have until now failed to attract the required long tail of editors. We believe that duplicate efforts will hurt the quality of available online information because the scarce number of active contributors is spread thinly over multiple resources. Furthermore, we hope Wikipedia will expand quality control measures in the future. Collaborations with other organizations should be set up to provide direct incentives for experts to contribute (such as coupling Wikipedia editing with article publication, with CME credits, or with funding).

In conclusion, we invite the medical community to join us in editing Wikipedia, with the goal of promoting health by providing readers worldwide with free access to reliable, understandable, and up-to-date health information.

## Abbreviations

CME: continuing medical education
PMID: PubMed Identifier
